# Introducing the Index of Caries Risk (ICR): A Comparative Study on a Novel Tool for Caries Risk Assessment in Pediatric Patients

**DOI:** 10.3390/children11101166

**Published:** 2024-09-25

**Authors:** Patrizia Lucchi, Alice Dina Nasuti, Giovanni Franciosi, Carlo Gaeta, Simone Grandini, Francesco Saverio Ludovichetti, Sergio Mazzoleni, Giulia Malvicini

**Affiliations:** 1 Department of Neurosciences—Dentistry Section, Padova University, 35128 Padova, Italy; patrizialucchi@marcorosa.it (P.L.); francesco.ludovichetti@unipd.it (F.S.L.); sergio.mazzoleni@unipd.it (S.M.); 2 Unit of Endodontics and Restorative Dentistry, Department of Medical Biotechnologies, University of Siena, 53100 Siena, Italy; alicedina.nasuti@student.unisi.it (A.D.N.); giofranciosi@gmail.com (G.F.); c.gaeta@unisi.it (C.G.); simone.grandini@unisi.it (S.G.)

**Keywords:** dental caries, pediatric dentistry, risk assessment

## Abstract

**Background:** The prevalence of dental caries presents a global public challenge, particularly in children. Traditional caries risk assessment tools like Cariogram are effective but often complex and resource intensive. The aim of the present study was to introduce, for the first time, a newly designed caries risk assessment (CRA) tool named Index of Caries Risk (ICR) and to evaluate its efficacy for pediatric patients. **Methods:** This observational study was conducted at the University Hospital of Siena (Italy), involving 55 children aged 6 to 12 years. Participants were assessed using both the newly developed ICR and the well-known Cariogram tool. The data were collected by two calibrated operators. The ICR was compared with the Cariogram tool, and a descriptive analysis and a Pearson correlation coefficient were performed. **Results:** Results indicated a strong positive correlation (R = 0.88, *p* < 0.01) between the two methods, with ICR simplifying the assessment process while maintaining efficacy. **Conclusions:** This study highlights the ICR’s potential to provide a practical, cost-effective alternative for routine caries risk assessment in pediatric dentistry. Despite its limitations, this research marks a preliminary investigation of a promising new CRA tool. Further research should focus on validating the ICR in the larger and more diverse pediatric population, as well as exploring its long-term effectiveness and its application in different clinical settings.

## 1. Introduction

Despite extensive research and the efforts to address dental caries, the prevalence of the disease remains a significant public health challenge for societies and governments on a global scale. In Italy, the prevalence of untreated caries in 2019 accounted for 36.1% and 29.6% in deciduous and permanent teeth, respectively [1,2,3].

The progression of dental caries can result in discomfort, pain, and ultimately tooth loss. Additionally, dental caries can cause loss of work or school days, adversely affecting productivity and educational outcomes, commonly estimated in productivity loss [4]. In 2015, global productivity losses due to dental diseases were estimated at $187.61 billion, with caries accounting for 12%, following severe tooth loss (67%) and severe periodontitis (21%) [5]. 

In light of the widespread prevalence of dental caries and its negative impact on general health and welfare, the importance to find effective strategies to prevent the onset and the progression the disease has become clear. This includes implementing measures and tools to identify patients at high risk of developing caries, such as the caries risk assessment (CRA) tool [6]. 

A CRA tool is a model to assist dental professionals in the identification of a patient at a high risk of developing caries and to address the detected risk factors [7,8,9]. Many CRA tools have been developed, typically using a scoring system to collect individual information and compute the cumulative effect of known risk factors [10,11].

Among the several CRA tools present in the literature, Cariogram is one of the methods that have demonstrated good clinical relevance in terms of ability to assess the risk and to aid the management of the risk [10].

This tool, based on a computer application, considers specific conditions such as diet, oral hygiene, caries experience, related diseases, and fluoride program. Once each factor is scored, the program generates a graphical representation that displays both the likelihood of avoiding caries and the risk of developing them [12,13].

Despite a large body of evidence supporting the clinical role of Cariogram, some limitations have been identified. For instance, some research suggests that the tool might lack in precision due to the absence of definitive thresholds for the risk categories [14]. Furthermore, a significant limitation of Cariogram is the need to collect salivary samples which can be challenging in pediatric dentistry due to the poor adherence to instructions. Moreover, salivary tests incur costs, and the correct use requires practice and experience in pediatric patients [15].

The promising results obtained from existing CRA tools, combined with the need for a more practical application in everyday dental practice, led to the development of a novel model designed to maintain efficacy while simplifying the evaluation process: the Index of Caries Risk (ICR).

The ICR is a newly designed pediatric CRA tool that was never used in previous studies. This tool examines the classical risk factors and indicators but also considers the unique characteristics and limitations of the population under examination. This approach ensures a quicker collection of patient information and includes an assessment of parental oral health status. Additionally, the ICR eliminates tests that require significant time and strict patient collaboration, which can be particularly challenging in pediatric patients.

The aim of this study is to introduce and explore the application of the ICR as a new method for assessing caries risk profiles, comparing its effectiveness and feasibility with the Cariogram.

## 2. Materials and Methods

### 2.1. Study Design

The present observational study was conducted from 7 March 2023 to 6 March 2024 in the departments of Pediatric Dentistry and Orthodontics of the University Hospital of Siena and approved by the local Ethics committee of the Area Vasta Sud-Est region of Tuscany (protocol number: 18993). 

The inclusion criteria of the study were as follows: (i) patients between the age of 6 and 12 years (ii) admitted for a first visit; (iii) the parents provided a consensus.

The exclusion criteria were (i) diseases, conditions, and medications that could interfere with caries onset (affecting salivation, creating chronic pH unbalance in the oral cavity, defective enamel structure); (ii) an ongoing or previous orthodontic treatment; (iii) inability or unwillingness of the legal guardian to give informed consent; (iv) and an inability of the patient and/or the legal guardian to communicate effectively in Italian.

Subjects fulfilling the previously described criteria were enrolled, and their caries risk was assessed through Cariogram and ICR.

The study was conducted in accordance with the Declaration of Helsinki. Before being included in the study, written informed consent was signed by all the study participant’s legal guardians after reading a patient information sheet.

### 2.2. Data Collection

Subjects were interviewed regarding medical conditions and pharmacological therapies. Patients with any systemic disease or under pharmacological therapies were excluded from the present study. Patients were also asked whether food was consumed preceding the visit. In case of food consumption 1 h prior to the visit, the salivary and buffer tests were performed at the second visit to exclude any risk of bias. 

The questionnaires (Tables S1 and S2) were administered by two calibrated operators (A.D.N. and G.M.) verbally both to the parents and the child, in Italian or English. The questions were wide-ranging on each topic, allowing the patient and the parent to elaborate and give more information; if the answer was not sufficient to find the proper score, more specific and detailed questions were made by the operator.

The clinical examination and assessment of disease indicators were performed with a WHO-CPI probe and a mirror in the presence of optimal artificial lighting. Clinical evaluation of carious lesions was performed by calibrated examiners. Calibration was performed using intra-oral digital photographs of the same tooth surfaces from the in vivo visual examination according to Christian et al., 2017 [16]. Kappa statistics were used to assess intra and inter-observer agreement using the statistical software STATA BE (version 17.1, StataCorp LP, College Station, TX, USA). 

Furthermore, examiners were calibrated with specific written guidelines on how to use a CRA form and assign the caries risk correctly. Afterwards, the examiners received a post-calibration test via a Qualtrics survey system using 22 pre-filled CRA forms of simulated patients. Inter-examiner reliability was calculated comparing the caries risk levels with the “gold standard” answer following instructions provided by Young et al., 2017 [17]. Kappa statistics were used to assess inter-observer agreement. 

### 2.3. Risk Assessment Using Cariogram

The relationship between different caries risk factors and the prediction of new caries through Cariogram was performed following the directions on the Malmö University Manual [12]; the scores were documented in a printed copy, Table S1, to ensure that the operator remained unaware of the outcomes during the assessment process.

All the children included in the study presented good general health and an absence of pathologies related to caries onset; therefore, for the item “related diseases”, the score was set at 0 for all children. 

A 3-day recall of meals and snacks was enquired to assess the diet frequency and diet content items. The evaluation of dmfs and DMFS is aimed at the assessment of caries experience and was scored from 0 (caries-free) to 3 [1,18]. The Silness and Löe plaque index [19] was used to assess the presence of plaque; the scores were assigned from 0 (extremely good oral hygiene, PS < 5%) to 3 (poor oral hygiene, PS > 50%).

The salivary secretion rate and buffer capacity tests were performed consecutively with the use of the Saliva Check Buffer^®^ kit by GC Europe (Leuven, Belgium). After instructing the patient, the paraffin-stimulated saliva secretion was collected for five minutes, and the rate was registered in ml/min. The sample collected was used to perform the strip test for the buffer capacity. The test results were interpreted in adherence with the instructions of the producer. 

In order to ensure the examination remained cost-effective and practical within the hospital setting, the mutans streptococci strip test was not performed in this study. This decision was made to minimize disruptions to patient visits. Clinical judgment was excluded from the registration, as in previous studies, to prevent any potential bias from the operator’s opinion on the outcomes. Only the risk factors and risk indicators were entered into the Cariogram form [13,20]. 

**Figure 1:**
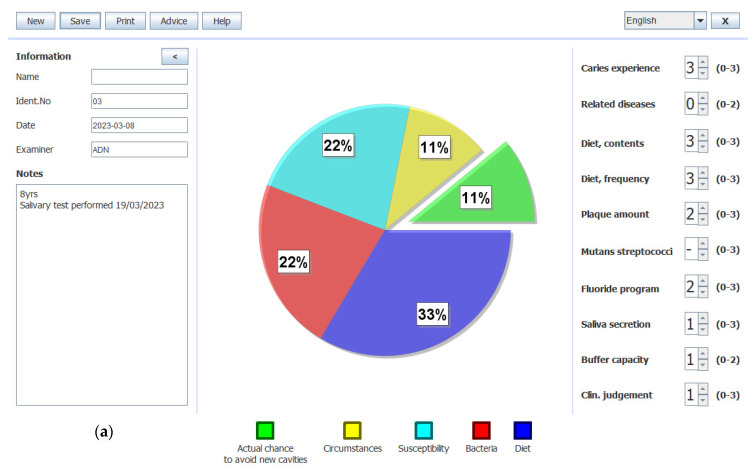

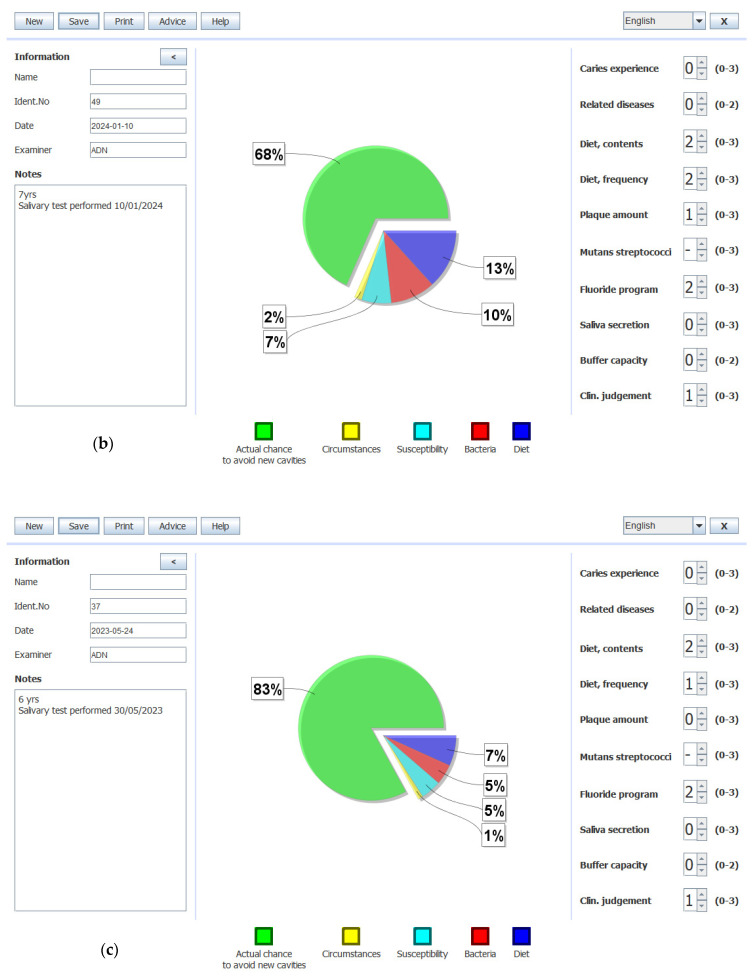
Examples of three Cariograms from the present study. (**a**) Cariogram illustrating a high risk for caries (11% chance of avoiding caries). (**b**) Cariogram illustrating a moderate risk for caries (68% chance of avoiding caries). (**c**) Cariogram illustrating a low risk for caries (83% chance of avoiding caries).

Therefore, Cariogram was performed through 8 items: caries experience, related diseases, diet content, diet frequency, plaque amount, fluoride program, saliva secretion and buffer capacity. According to the score, patients identified as having low (<25%), moderate (25–75), or high (>75%) chances to avoid caries [12,14]. For the aim of this study, the value of risk was recorded. To ensure consistency with the aim of this study, which considers caries risk, the Cariogram score was adjusted accordingly. The adjustment involved replacing the Cariogram score of chances of avoiding caries with its complementary value (to 100%), enabling the classification of patients into categories of a low, moderate, or high risk of developing caries. Examples are shown in Figure 1. For instance, if the computed Cariogram value was 33%, it was replaced with 67% (100 minus 33) for analysis and ranking purposes.

### 2.4. Risk Assessment Using ICR

The assessment of the risk profile according to the ICR CRA tool was conducted following the questionnaire, available as Table S2.

Similar to the assessment for the Cariogram items, the 3-day recall was reviewed to assess the appropriate score to assign to the eating habits. The oral hygiene practice and exposure to fluoride were recorded after the operator’s survey.

The dental history of the parents was evaluated retrospectively by reviewing existing clinical records. If these records were unavailable or outdated, parents were asked about any current pain or discomfort.

During the clinical examination, children’s caries experience and oral hygiene status were identified following the direction of the CRA tool; therefore, DMFT and dmft statuses were recorded.

The salivary pH value was assessed through the litmus test strips available in the Saliva Check Buffer^®^ kit by GC. The strips were placed on the tongue and left for 10–15 s in the mouth with the lips sealed, and, afterwards, their color was matched with the reference chart provided in the kit’s instructions.

The ICR was determined though the sum of the 8 items’ scores: diet content, eating frequency, hygiene habits, fluoride exposure, family susceptibility, caries experience, oral hygiene status, and pH test. After computing the sum score, the patients were categorized as having low (0–5), moderate (6–10), high (11–15), or very high risk (16–21) of caries risk.

### 2.5. Practical Issues and Additional Considerations

(i) Due to the hospital’s protocol of a mouthwash rinse with Chlorhexidine before each visit, the salivary tests were performed before the question items. In case it was not possible, due to time or possible influences of other factors, the tests were performed at a second visit.
(ii) The size was influenced by a dropout of 14 patients caused by their failure to attend a second visit to perform the salivary tests.
(iii) Whenever the answers of the child and guardian were inconsistent, more questions were made to investigate which score was most appropriate.

### 2.6. Statistical Analysis

The sample size for this observational study was determined based on preliminary data indicating a strong correlation between the Index of Caries Risk (ICR) and the established Cariogram tool. 

Given a significance level (α) of 0.05 and a desired power (1 − β) of 0.80, we anticipated a strong correlation coefficient (R) of 0.7 based on preliminary findings. To account for potential dropouts and variability, the final sample size was adjusted to include 55 children aged 6 to 12 years. This sample size ensures robust statistical power to detect the expected correlation between the ICR and the Cariogram tool, thereby validating the effectiveness of the ICR in caries risk assessment.

Statistical analysis was performed using the analysis ToolPak add-ins on Microsoft Excel (Version 16.0; Microsoft Corporation, Redmond, WA, USA, 2023). A descriptive analysis was performed for each CRA tool. Pearson’s correlation was conducted on the quantitative results of each CRA tool.

## 3. Results

A total of 55 pediatric patients were included in the present study (28 female and 27 male) with a mean age of 8.38 (SD = 1.99). Results of the caries risk assessments revealed that the mean score (M) of the patients on the Cariogram was M = 52.0% (SD = 28.0%), ranging from 12% to 96%. Table 1 shows that 35% (n = 19) of all children in the study were identified as having a high caries risk, 38% (n = 21) had moderate caries risk, and 27% (n = 15) had low caries risk. On the ICR, the mean score (M) of the patients was M = 9.3 (SD = 4.0), with a range of 3–20. Table 2 shows that 13% (n = 7) of all children in the study were identified as having a very high risk of caries, 22% (n = 12) were identified as having a high caries risk, 51% (n = 28) were identified as having moderate risk, and 15% (n = 8) fell in the low-risk category. The distribution of scores for each caries-related factor was summarized in the Table 3, which includes the number of children assigned to each score and the corresponding percentage. Results of the caries risk assessed through the two tools were merged as shown in Table 4. 

Calibration for clinical evaluation of carious lesions resulted in kappa = 0.76 (95% CI: 0.69–0.79; *p* < 0.05) for the first examiner and kappa = 0.79 (95% CI: 0.71–0.84; *p* < 0.05) for the second examiner. Inter-examiner agreement resulted to be good (kappa = 0.74, 95% CI: 0.69–0.81; *p* < 0.05). Regarding the use of CRA tool, the strength of agreement resulted to be good, with kappa = 0.72, 95% (CI: 0.67–0.81; *p* < 0.05). 

**Figure 2:**
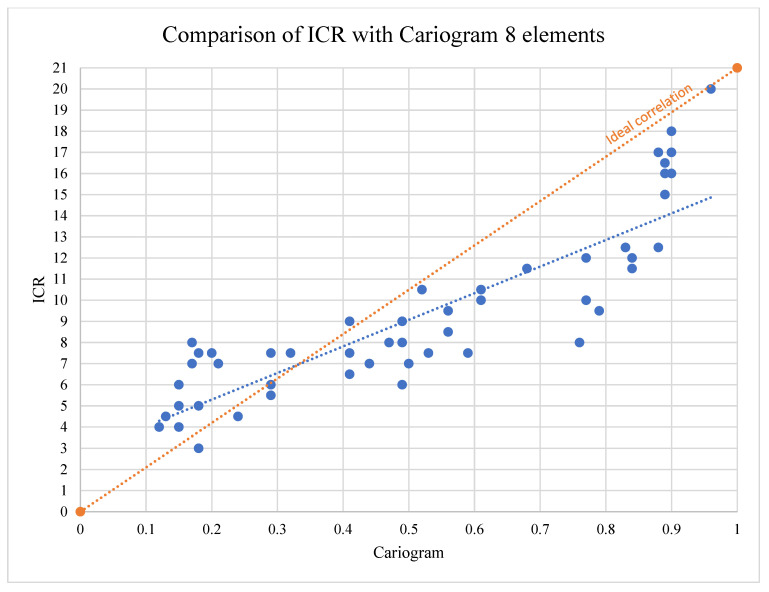
Comparison of ICR and Cariogram. The blue line represents the linear correlation of the data.

The Pearson’s correlation coefficient was calculated to evaluate the relationship between the risk values produced by the two risk assessment tools. To ease the interpretation of the graphical representation (Figure 2) for the Cariogram, the corresponding value of the risk was plotted. The analysis revealed a strong positive linear association, with a correlation coefficient of R = 0.88 (*p* < 0.01).

## 4. Discussion

This study explored the ability of ICR to identify the caries risk profile in a sample of 55 children aged 6 to 12 years, based on the concordance on the risk assessment conducted with the CRA tool under investigation and the Cariogram.

The results of the ICR CRA tool were collected with caries-related factors in Table 2. Despite this, it was not possible to compare the results with other population samples due to the absence of previously available data collected with this novel CRA tool. Table 2 highlights two important observations. Notably, despite recommendations from the American Academy of Pediatric Dentistry (2016, 2012) [21,22] and the European Association of Pediatric Dentistry (2009) [23] that children should be assisted and supervised in oral hygiene practices, the data indicate a trend of unsupervised toothbrushing among children. Conversely, it is encouraging that 98.2% of children have access to at least one source of fluoride, primarily through fluoride toothpaste. This highlights the positive effect that policies have achieved in raising awareness on the role of fluoride in caries progression control [23,24].

Divergent patterns in caries risk distribution were evident when comparing the two CRA tools. Notably, ICR assessment showed that a significant portion of the children (51%, n = 28) fell into the moderate-risk category. Conversely, the Cariogram results revealed a more balanced distribution across the risk categories, with a slightly elevated proportion (38%, n = 21) falling within the moderate-risk bracket. These results are not entirely aligned with the outcomes observed in earlier studies conducted in a pediatric population of children from a different Italian region [18,25].

It is crucial to recognize that these discrepancies are significantly influenced by the risk categories reported and relative thresholds established for the Cariogram. Unlike other clinical risk assessment tools, there isn’t a universally accepted set of thresholds for the CRA tool. Several thresholds have been employed across different studies [13,26,27], highlighting the lack of clarity and the resultant allocation biases [14]. Therefore, to address this variability, the quantitative results of the CRA tools were compared. 

The comparison analysis between ICR with Cariogram reveals a high degree of concordance in assessing caries risk between the two tools. This finding suggests that the ICR performs comparably well to the Cariogram in evaluating the risk of developing new caries in the analyzed sample.

Despite the high degree of concordance, the correlation between the two CRA tools shows a slope that deviates significantly from the slope of the ideal correlation (Figure 2). 

A noteworthy aspect is the difference in methods used by the two CRA tools to estimate the risk. Whereas Cariogram relies on a complex formula involving multiple conditional statements [12,13], the ICR risk results is a straightforward sum of values assigned to individual risk factors.

A large body of evidence supports the clinical role of the Cariogram in identifying patient risk and aiding in the management of risk factors [10]. Moreover, the interactive PC program shows patients how the change of one single habit could reduce their risk of developing caries. This aspect could significantly improve oral health awareness. However, the specific impact of the interactive program on oral health awareness remains unexplored.

In the Cariogram, family predisposition to caries and attitudes toward oral health are indirectly considered, together with socio-economic factors, under the “clinical judgment” category (Table 5) [12]. In contrast, the ICR CRA tool provides a distinct evaluation for this factor. This approach is particularly relevant in pediatric patients, where a child’s oral health is strongly influenced by parental decisions and adherence to recommended behavioral changes [28]. Indeed, the Caries Management by Risk Assessment (CAMBRA) tool considers the presence of dental decay in caregivers or siblings for the risk assessment in patients aged 0 to 5 years [29], though this factor is not included in the CRA tool for patients aged 6 through adulthood [30,31]. Therefore, the ICR’s inclusion of an assessment of the parents’ oral health status is a necessary addition to accurately assessing a child’s caries risk (Table 5).

Nonetheless, the challenge of gathering data samples from children under 12 years old, particularly concerning paraffin-stimulated salivary secretion is often underscored. Although several studies suggest the feasibility of performing the CRA tool without salivary tests [32,33], such approaches have yielded inconsistent results [34]. 

A prior investigation study conducted on a sample of Italians aged 7 to 9 years omitted the collection of data on saliva secretion rate, asserting that hyposalivation is not common in younger children and, when present, is often associated with systemic disease [18]. However, the analysis of collected data revealed that only 27 children (49.1%) exhibited a normal salivation rate exceeding 1.1 mL/min. The remaining 28 children displayed low secretory rate: 25 children (45.5%) ranged between 0.9 and 1.1 mL/min, while 3 children (5.5%) had rates between 0.5 and 0.9 mL/min. Notably, none of the children exhibited severely low secretion rates or xerostomia. This discrepancy might be attributed to the challenges younger children face in accurately adhering to instructions for the saliva collection.

On the other hand, when it comes to the ICR CRA tool, the pH salivary test stands out as the sole component that requires the use of an instrument not commonly found in dental clinics: a litmus test. Yet, this tool is easily accessible and cost-effective. Furthermore, the accompanying instructions are concise and straightforward, demanding minimal effort from the patient and only 10 to 15 s for the data collection. Table 5 summarizes the different items present in the CRA tools: Cariogram, CAMBRA (6 through adulthood) [30], and ICR. Thus, the advantage that ICR offers in the dental clinic for routine caries risk assessment in children is evident in terms of its user-friendliness, time efficiency, and cost-effectiveness.

The present study must be seen in light of some limitations. First, the small sample that was significantly influenced by patient compliance with attending a second visit for saliva data collection. Additionally, the increasing number of patients exhibiting signs of Molar incisor hypomineralization (MIH), associated with increased caries experience, led to their exclusion from the study [35]. While a larger sample size would enhance the detection of marginal cases, our primary objective was to lay the groundwork for the ICR CRA tool. Similar studies with initial smaller sample size have been crucial in the preliminary phases of medical diagnostic tool development, establishing early efficacy and feasibility [36]. For example, initial research on various tools for the caries risk assessment often begins with limited samples to identify promising outcomes before scaling up [37,38]. The exclusion criteria themselves also create a limitation, as they restrict the study population sample. For instance, the decision to include only patients aged 6 to 12 years was based on two primary considerations. First, dental care access within the Italian public healthcare system is mainly used by children from 4 to 5 years of age, as evidenced by the study of Petti et al., 2006 that indicates that dental visits are more frequent in this age group [39]. Second, collecting salivary samples for quantitative analysis from patients aged 0 to 5 years presents challenges. Children in this younger age group often exhibit limited cooperation during procedures necessary for accurate sample collection, potentially compromising data reliability. These challenges are also noted in guidelines from the American Academy of Pediatric Dentistry (AAPD) [40] and Ramos-Gomez et al., 2007 [41], which emphasize age-specific considerations in managing caries risk and data collection. Future research should focus on broader patient samples, including those with conditions currently excluded to assess the effect these factors may have on the performance of the ICR tool.

Second, the inclusion of patients solely from a single institution undermines the generalizability of the study findings. Conducting a multi-center study, potentially incorporating samples from private dental clinics, could enhance this aspect.

Lastly, a risk assessment tool should serve two primary functions: identifying individuals at risk (prediction model) and identifying risk factors (risk model) to guide clinical decision-making [42]. While the Cariogram CRA tool, according to Petersson and Bratthall, embodies characteristics of both a risk and prediction [12,13], the data collected in this study suggest that the ICR CRA tool operates solely as a prediction model. Conducting a prospective study to evaluate actual caries outcomes could provide further insights into the role of the ICR tool as a risk model.

## 5. Conclusions

This study’s findings suggest that the ICR CRA tool exhibits comparable outcomes to the Cariogram in dental risk assessment.

Given the crucial role of caries risk assessment in preventive dentistry, it’s vital to continue researching CRA tools. These tools should accurately identify risk categories and factors while also being efficient and cost-effective. Despite its limitations, this research marks a preliminary investigation of a promising new CRA tool. Further studies with larger samples, varied contexts, and longitudinal assessment of caries outcomes could reveal the full potential of ICR as a valuable tool in everyday clinical practice.

## Figures and Tables

**Table 1:** Assessment of caries risk using Cariogram tool (N = 55).

Risk	Score ^1^	*n*	%
High	0–25%	19	35
Moderate	26–75%	21	38
Low	76–100%	15	27

^1^ The thresholds refer to the risk values, therefore the complementary value of the “chances to avoid caries”.

**Table 2:** Assessment of caries risk using ICR tool (N = 55).

Risk	Score	*n*	%
Very High	16–21	7	13
High	11–15	12	22
Moderate	6–10	28	51
Low	0–5	8	15

**Table 3:** Results of the ICR for each caries-related factor.

Factor		Number of Children	Percentage of Group (%)
Diet content	low consumption of sugars	2	3.6
	moderate consumption of sugars	19	34.5
	high consumption of sugars	22	40.0
	severe consumption of sugars	12	21.8
Diet frequency	0–3 meals	3	5.5
	4–5 meals	35	63.6
	6–7 meals	10	18.2
	more than 7 meals	7	12.7
Oral hygiene habits	brushing 3 times a day performed by a parent	2	3.6
	brushing 2 times a day performed by a parent	3	5.5
	brushing 2 times a day with supervision	10	18.2
	brushing 1–2 times a day without supervision/no brushing	40	72.7
Fluoride program	fluoride toothpaste + fluoride mouthwash + fluoride os (in the past)	0	0.0
	fluoride toothpaste + fluoride os (in the past)	0	0.0
	fluoride toothpaste	54	98.2
	absence of fluoride program	1	1.8
Familiarity with caries	mother − father −	16	29.1
	mother + father −	11	20.0
	mother ++ father +/−	16	29.1
	mother ++ father ++	12	21.8
Caries experience	absence of caries + no old restorations + absence of teeth missing due to caries	22	40.0
	1 or 2 caries (or restorations or teeth missing due to caries)	8	14.5
	2 to 4 caries (or restorations or teeth missing due to caries)	7	12.7
	4 or more caries (or restorations or teeth missing due to caries)	18	32.7
Oral hygiene status	no plaque	8	14.5
	plaque without bleeding	26	47.3
	plaque with bleeding, calculus, marginal gingivitis	14	25.5
	plaque with bleeding, calculus, gingivitis in multiple sites	7	12.7
pH test	alkaline pH (>7)	34	61.8
	neutral pH (7)	20	36.4
	acidic pH (6.5–5.5)	0	0.0
	critical pH (<5.5)	1	1.8

+ signals the presence of caries susceptibility; − signals the absence of caries susceptibility; ++ signals a high caries susceptibility.

**Table 4:** Caries risk assessed merged.

			Cariogram	
	Risk	Low	Moderate	High
ICR	Low	8		
	Moderate	7	18	3
	High		3	9
	Very high			7

**Table 5:** Summary of the main differences of CRA tools.

		Cariogram	CAMBRA (6 through Adulthood)	Index of Caries Risk
Host				
Saliva quality/salivary quantity	Salivary buffer test	X		
	pH salivary test			X
	Salivary secretion rate	X	X	
	Hyposalivatory medications		X	
Oral hygiene habits	Brushing habits		X	X
	Parental involvement in brushing			X
	Use of additional non-fluoride dental products		X	
Fluoride exposure	Fluoridated water		X	
	Fluoridated toothpaste	X	X	X
	Additional fluoride intake	X	X	X
Diet/frequency of snacking/sugary drinks	Carbohydrates intake	X		X
	Snacking frequency	X	X	X
	Sugary drink			X
General health conditions/eating disorders		X	X	
Parents’ oral health/caregiver oral health		X *		X
Socio-demographic status		X *		
**Dental Plaque**				
Presence of plaque		X	X	X
Bacterial substrate	Mutans streptococci count	X		
	Lactobacillus count	X **		
	Cariogenic bacteria quantity		X ^†^	
**Tooth**				
Unusual tooth morphology/deep pit and fissures			X	
Orthodontic appliances/space maintainers			X	
Caries experience	White spots		X	
	dmft/DMFT			X
	Lesion progression		X	
	Assessment within the age group	X		
	Radiographic assessment		X	
Risk categories		Low, Moderate, High or Low, Low/Moderate, Moderate/High High	Low, Moderate, High, Extreme	Low, Moderate, High, Very High

* Considered in the “Clinical judgment” item. ** in support to “diet contents” item. † currently not available [31].

## Data Availability

The original contributions presented in the study are included in the article/Supplementary Material, further inquiries can be directed to the corresponding author.

## Supplementary Materials

The following supporting information can be downloaded at: https://www.mdpi.com/article/10.3390/children11101166/s1, Table S1: Cariogram Data Collection Table. The table used for the data collection in the Cariogram assessment. The table is a printed copy of the original questionnaire format, which helped to ensure that the operator remained blinded to the results; Table S2: Index of Caries Risk (ICR) Data Collection Table. The table used for data collection in the assessment through Index of Caries Risk (ICR). The format allows easy data entry and subsequent analysis, contributing to the study’s objective of comparing the novel ICR tool with the Cariogram method.
