# Supplementary material for: Introducing the Index of Caries Risk (ICR): A Comparative Study on a Novel Tool for Caries Risk Assessment in Pediatric Patients
Source: Children (Basel). 2024 Sep 25;11(10):1166. doi: 10.3390/children11101166 (PMC11505636; doi:10.3390/children11101166)
Supplement: Supplementary file 1 [file children-11-01166-s001.zip › Table S1.pdf]

CARIOGRAM

Patient: **Age:** **Date:**

Values	0	1	2	3
Caries experience	Completely caries-free, no previous fillings, no cavities or missing teeth due to caries	Better status than normal, for that age group in that area	Normal status for that age group	Worse status than normal, for that age group, or several new caries lesions the last year
Related Diseases	No general diseases of importance related to dental caries	Mild degree disease/conditions indirectly influencing caries		
Diet content	Extremely good diet with caries-inducing carbohydrate on a very low level	Appropriate diet. Sugars or other caries-inducing carbohydrate on a low level	Diet with relatively high content of sugars or other caries-inducing carbohydrate	Inappropriate diet. High intake of sugars or other caries-inducing carbohydrate
Diet frequency	max 3 meals daily	4-5 meals daily	6-7 meals daily	More than 7 meals daily
Plaque amount	Extremely good oral hygiene (PS<5%)	Good oral hygiene (PS 5-20%)	Less than good oral hygiene (PS 20-50%)	Poor oral hygiene (PS >50%)
Fluoride program (exclude water fluoridation)	Fluoride toothpaste plus constant additional fluoride measures	Fluoride toothpaste plus inadequate (infrequent) additional fluoride measures	Fluoride toothpaste only	Not using fluoride toothpaste or other fluoride measures
Saliva secretion	Normal	Low (0.9-1.1ml/min)	Low (0.5-0.9ml/min)	Very low, xerostomia (<0.5ml/min)
Buffer capacity	Adequate Dentobuff blue pH>6.0	Reduced Dentobuff green pH 4.5-5.5	Low Dentobuff yellow pH<4.0	
Clinical judgement (caries situation and social factors)	More positive impression	Normal impression	More negative impression	Very bad impression
