# Supplementary material for: Introducing the Index of Caries Risk (ICR): A Comparative Study on a Novel Tool for Caries Risk Assessment in Pediatric Patients
Source: Children (Basel). 2024 Sep 25;11(10):1166. doi: 10.3390/children11101166 (PMC11505636; doi:10.3390/children11101166)
Supplement: Supplementary file 1 [file children-11-01166-s001.zip › Table S2.pdf]

ICR index caries risk

Name and Surname Date

**What type of diet does the patient follow?**	Low sugar (carbohydrates with brushing, no sugary drinks)	0	Medium sugar (carbohydrates + brushing, occasional sugary drinks	1	High sugar (carbohydrates without brushing, occasional sugary drinks)	2	Severe sugar (carbohydrates without brushing, frequent sugary drinks)	3
**How many meals does the patient consume per day?**	0 – 3 meals per day	0	4 – 5 meals per day	2	6 – 7 meals per day	3	More than 7 meals per day	4
**What type of oral hygiene routine does the patient follow?**	Brushing 3 times a day, active home biofilm control with parents in the morning and evening	0	Brushing 2 times a day, active home biofilm control with parents in the morning or evening	0.5	Brushing 2 times a day + supervision by parents in the morning or evening	1	Brushing 1 -2 times a day without supervision / no brushing	2
**What type of dental fluoride product does the patient use?**	Fluoridated toothpaste + fluoridated mouthwash + oral fluoride supplements	0	Fluoridated toothpaste + oral fluoride supplements	0	Fluoridated toothpaste	0	No fluoridated toothpaste	1
**What is the caries susceptibility of the parents?**	Mother - / Father -	0	Mother + / Fater -	0.5	Mother ++ / Father +/-	1	Mother ++ / Father ++	2
**Caries experience (DMFT)**	No caries + no fillings + no missing teeth due to caries (DMFT =0)	0	Presence of 1 or 2 caries (or fillings or missing teeth due to caries) (DMFT=1-2)	1	Presence of 2 to 4 caries (or fillings, or missing teeth due to caries) (DMFT=2-4)	2	Presence of 4 or more caries (or fillings, or missing teeth due to caries) (DMFT>4)	3
**Oral hygiene level**	No plaque	0	Plaque without bleeding	0.5	Plaque with bleeding, calculus, marginal gingivitis	1	Plaque with bleeding, calculus, marginal gingivitis in multiple sites	3
**pH test**	Alkaline pH (>7)	0	Neutral pH (7)	0	Acidic pH (between 6.5 and 5.5)	1	Critical pH (<5.5)	3
**Total per category**		0		5.5		11		21
**RISK INDEX**								37.5

**Caries risk**	MILD		MODERATE		HIGH		VERY HIGH	
Caries risk	0	5	6	10	11	15	16	21
